# Evaluating Preferences of Hospitalized Diabetes Patients for Hospital-Wide Glycemic Control Programme: A Discrete Choice Experiment

**DOI:** 10.1155/2024/2552658

**Published:** 2024-09-06

**Authors:** Jing Dai, Ting He, Xiaodie He, Huaying Li, Lintong Li, Jie Sun, Jie Pan, Cheng Ji

**Affiliations:** ^1^ Department of Pharmacy The Second Affiliated Hospital of Soochow University, Suzhou, China; ^2^ Department of Pharmacy China Pharmaceutical University Nanjing Drum Tower Hospital, Nanjing, China; ^3^ Department of Endocrinology Endocrine and Metabolic Disease Medical Center Nanjing Drum Tower Hospital Clinical College of Nanjing University of Chinese Medicine, Nanjing, China; ^4^ Department of Pharmacy Xishanqiao Community Health Service Center, Nanjing, China; ^5^ Department of Pharmacy Nanjing Drum Tower Hospital Affiliated Hospital of Medical School Nanjing University, Nanjing, China; ^6^ Department of Endocrinology Nanjing Drum Tower Hospital Affiliated Hospital of Medical School Nanjing University, Nanjing, China

**Keywords:** DCE, diabetes, glycemic control, hospitalization, patient care management, patient preference

## Abstract

**Background:** Effective glycemic control is crucial for hospitalized patients, leading to benefits such as shorter hospital stays and reduced postoperative infection rates. While previous studies have emphasized the effectiveness of multidisciplinary collaborative stewardship for hospital-wide hyperglycemia management, patient perspectives and preferences have not been adequately considered.

**Objective:** To identify factors influencing treatment preferences of Chinese hospitalized diabetes patients using discrete choice experiments (DCEs) and provide practical insights for the construction of a hospital-wide glycemic control programme.

**Methods:** A face-to-face survey was conducted among diabetes patients admitted to nonendocrine departments in a tertiary hospital in Nanjing, China. The attributes and levels were determined based on DCE principles, and a conditional logit model was used to quantify patients' preferences.

**Results:** A total of 157 respondents were analyzed. Antihyperglycemic effectiveness, healthcare providers, treatment regimen, monitoring frequency, and adverse reactions were the five attributes that significantly influenced patient preference (*p* < 0.05). Notably, an 80% glycemic control rate (*β* = 2.009) and a multidisciplinary management team involving clinical pharmacists (*β* = 1.346) had the greatest impact. Negative effects were observed for hypoglycemia (*β* = −1.008), insulin pump use (*β* = −0.746), and frequent glucose monitoring (*β* = −0.523). Female patients exhibited higher concern for healthcare providers (*β* = 1.172) compared to males. Younger and shorter-course patients prioritized antihyperglycemic effectiveness (*β* = 3.330, *β* = 1.510), while older patients preferred multidisciplinary management (*β* = 1.186) and opposed increased monitoring frequency (*β* = −0.703). Patients with higher educational backgrounds showed greater acceptance of continuous glucose monitoring (*β* = 1.983), and those with higher annual income placed more emphasis on glycemic control rate.

**Conclusion:** Treatment preferences of hospitalized diabetes patients are mainly influenced by antihyperglycemic effectiveness, adverse reactions, healthcare providers, and individual characteristics. Comprehensive consideration and an individualized therapy strategy should be given when constructing a hospital-wide glycemic control programme.

## 1. Introduction

Type 2 diabetes (T2DM) is a chronic and highly heterogeneous progressive disease characterized by both inherited and acquired insulin resistance, along with disturbances in qualitative and quantitative insulin secretion. Globally, the incidence of T2DM has been on a continuous rise due to factors such as population aging, improvements in economic conditions leading to changes in dietary habits and modes of transportation, and an increasing prevalence of overweight and obesity. According to data from the International Diabetes Federation (IDF), approximately 515 million adults worldwide were affected by T2DM as of 2021, with an estimated projection of reaching 704 million patients by 2045 [1]. Notably, China has the highest number of diabetes patients in the world [1], imposing substantial burdens on affected individuals, their families, the healthcare system, and society as a whole.

Effective antihyperglycemic treatment can slow the progression of T2DM and prevent various complications, including diabetic nephropathy, retinopathy, and peripheral arterial disease [2]. However, glycemic control among Chinese T2DM patients remains suboptimal. Cross-sectional studies conducted in different regions have shown that the proportion of T2DM patients achieving adequate glycemic control, defined as a recommended HbA1c level of less than 7.0% according to clinical guidelines [3–5], ranges from approximately 22.45% to 44.46% [6–10]. Among these patients, those admitted to nonendocrinology departments due to other illnesses often experience longer hospital stays, higher healthcare expenditures, increased rates of hospital-acquired infections and mortality, and suboptimal response to antihyperglycemic treatments. This may be attributed to glycemic fluctuations caused by comorbidities or concurrent medications, as well as the lack of a specialized hyperglycemic management team [11].

The increasing number of hospitalized T2DM patients poses challenges for traditional consultations and self-management programmes in nonendocrine departments. Consequently, there has been a growing emphasis on multidisciplinary collaborative stewardship for hospital-wide glycemic control among healthcare providers. Currently, several institutions in China have established an internal glycemic control programme, forming specialized teams consisting of endocrinologists, nurses, clinical pharmacists, dietitians, and exercise specialists, utilizing the hospital's electronic glucose monitoring system, to provide comprehensive, continuous, and individualized management for all hospitalized hyperglycemic patients [12–14]. While several studies have examined the clinical effects of this innovative programme, there has been limited analysis regarding the perspectives and preferences of the patients themselves.

Treatment willingness and preference denote the informed decisions made by patients subsequent to assessing the anticipated advantages and drawbacks of diverse treatment modalities, encompassing efficacy, adverse effects, and expenses associated with each option. With the progressive refinement of patient-centered strategies in diabetes management, the acquisition of data pertaining to patients' experiences, perspectives, and inclinations throughout the clinical intervention process and their adept integration into patients' treatment regimens and care plans has assumed heightened significance. Both the American Diabetes Association (ADA) and the European Association for the Study of Diabetes (EASD) have advocated for the active involvement of patients in all treatment determinations within the realm of diabetes management, emphasizing the primacy of patients' preferences, requirements, and ethical values [15]. Consequently, in the endeavor to establish and enhance a novel hospital-wide glycemic control initiative, due consideration and reverence for patient preferences are imperative, aiming to shift patients from being mere recipients of treatment to engaged participants therein, thereby fostering enhanced adherence and glycemic regulation [16, 17].

At present, the quantitative assessment of patient preferences is commonly categorized into two main methods: stated preference (SP) and revealed preference (RP). SP entails analyzing feedback obtained from respondents under hypothetical scenarios, while RP involves examining actual behaviors observed in real-life situations. Discrete choice experiment (DCE), a subtype of the SP method, has been widely utilized in preference studies within the healthcare domain due to its unique advantages in experimental design and data analysis methods [18]. In simple terms, researchers first need to determine research objectives and a series of attributes and relevant levels that constitute the objective. Attributes may include features such as effectiveness, safety, or mode of administration of pharmaceuticals, biological treatments, or medical devices, while attribute levels describe the possible values, outcomes, interventions, or technologies associated with each attribute. Subsequently, all possible attributes and levels are combined to create several alternative hypothetical scenarios, which are then presented to respondents in the form of a survey questionnaire. Respondents weigh the importance of different attributes and levels, choosing the programme they consider to be optimal. Researchers employ statistical models to qualitatively and quantitatively analyze patients' treatment preferences. A number of similar DCE studies have been conducted previously, focusing on different kinds of antihyperglycemic drugs, glucose monitoring technologies, or Type 1 diabetes screening strategies [19–22]. To date, no relevant DCE research has been conducted to investigate preferences for hospital-wide glycemic control programme among Chinese T2DM patients.

Therefore, this study is aimed at using DCE to explore the preferences of T2DM patients in nonendocrine departments regarding in-hospital glucose management patterns, as well as to identify the key attributes and personal characteristics that influence their choices. The results of this research will provide practical insights for clinical decision-makers and help achieve patient-centered and shared decision-making (SDM) healthcare.

## 2. Material and Methods

This study adopted a cross-sectional study design conducted at Nanjing Drum Tower Hospital, Affiliated Hospital of Medical School, Nanjing University, Nanjing, China. The research protocol received approval from the hospital's Medical Ethics Committee (grant number: 2020-263-02) and conforms to the provisions of the Declaration of Helsinki. The research methodology and procedures adhered to the guidance provided by the ISPOR task force report on conducting a well-designed DCE [18]. To begin with, we identified the primary attributes and levels of preference through an extensive literature review, expert consultations, and patient interviews. Subsequently, we employed the orthogonal experimental design (OED) method to combine various attributes and different levels, thereby constructing choice sets for the DCE questionnaires. Finally, data were collected through face-to-face interviews and analyzed using appropriate data processing models.

### 2.1. Attributes and Levels

Determining the attributes and levels stands as the pivotal initial phase in executing a DCE. Generally, two key considerations emerge: (1) relevance: the selected attributes and levels must closely align with the research focus (hospital-wide glycemic control programme) and possess substantive pertinence to the respondents (hospitalized hyperglycemic patients) while remaining readily comprehensible. (2) Quantity: the quantity of attributes and levels must strike a balance; an excessive number may engender overly intricate questionnaire tasks, potentially prompting respondents to make choices impulsively, thereby compromising the interpretive capacity of DCE results. Conversely, an inadequate number may overlook attributes of relative importance to respondents, thus inadequately capturing preferences.

Presently, qualitative research methodologies (including literature review, expert consultation, and patient interviews) are esteemed as the “gold standard” for delineating DCE attributes [18]. In this study, we conducted a thorough search of recent literature concerning treatment preferences among diabetes patients in major medical databases such as PubMed, Embase, and Cochrane. A systematic review by Liu et al. [23] examined 30 DCE studies on treatment preferences of T2DM, identifying three commonly considered attributes: antihyperglycemic effectiveness, treatment regimen (administration route, timing, and frequency), and adverse drug reaction (hypoglycemia, gastrointestinal reactions, and weight changes). Building upon this research, our study incorporates the distinctive aspects of in-hospital hyperglycemia management and introduces three additional attributes: healthcare providers, monitoring frequency, and management cost, with their respective levels delineated. This comprehensive approach leads to the formulation of a preliminary attribute set tailored specifically to the research objectives and the characteristics of the target population. Then a panel of six experts was convened for face-to-face consultations, including deputy chief physicians, deputy chief nurses, clinical pharmacists with more than 5 years of experience in endocrinology, and a university professor familiar with DCE. These experts determined a set of six characteristic attributes, namely, antihyperglycemic effectiveness, treatment regimen, healthcare providers, glucose monitoring frequency, adverse drug reaction, and management cost.  Table 1 lists the included six attributes, 17 levels, introduction to patients, and their assignments. Here is a detailed explanation of each attribute.

#### 2.1.1. Antihyperglycemic Effectiveness

Previous research has shown that treatment effectiveness is typically the primary outcome of interest for researchers and a critical attribute influencing patient adherence. For short-term in-hospital glycemic control, HbA1c < 7.0% may not be an appropriate efficacy indicator. The expert consensus on hyperglycemia management in hospitalized patients in China recommends establishing individualized control targets of blood glucose concentrations for patients with different conditions [24]. For instance, stricter targets (fasting blood glucose 4.4~6.1 mmol/L, postprandial 2-h blood glucose 6.1~7.8 mmol/L) are suitable for newly diagnosed and younger patients with low hypoglycemia risk. Moderate targets (fasting blood glucose 6.1~7.8 mmol/L, postprandial 2-h blood glucose 7.8~10.0 mmol/L) are appropriate for elective surgery patients or those using glucocorticoids. More lenient targets (fasting blood glucose 7.8~10.0 mmol/L, postprandial 2-h blood glucose 7.8~13.9 mmol/L) are suitable for patients hospitalized for cardiovascular or cerebrovascular diseases, those aged 75 years and above, and those in critical care. In this study, the last blood glucose monitoring result before discharge serves as the criterion for assessment. Antihyperglycemic effectiveness is quantified as the proportion of patients with adequate glycemic control among all T2DM inpatients and is divided into three levels: low (40%), moderate (60%), and high (80%).

#### 2.1.2. Treatment Regimen

The expert consensus recommends subcutaneous insulin injections or insulin pumps for hyperglycemic patients hospitalized in nonendocrine departments; for those with relatively stable clinical conditions, they can also continue using oral antihyperglycemic drugs taken prior to admission [24]. Therefore, this study considers three levels for the treatment regimen attribute: oral antihyperglycemic drugs, multiple daily insulin injections, and subcutaneous insulin pumps.

#### 2.1.3. Monitoring Frequency

Currently, the glucose monitoring methods for hospitalized patients are mainly bedside capillary blood monitoring and continuous glucose monitoring (CGM). For the former, the patient's pain experience increases with the increase of monitoring times, which can affect patient treatment satisfaction. In contrast, CGM can avoid repeated finger pricks and reduce patient discomfort by implanting the needle subcutaneously. Therefore, this study sets three levels within this attribute: 4 times/day, 7 times/day, and CGM.

#### 2.1.4. Adverse Drug Reaction

During the treatment process, patients often change or discontinue their medications without approval due to adverse drug reactions, leading to poor glycemic control. This study summarized the common adverse reactions as three levels: gastrointestinal reactions, allergic reactions (skin pruritus, etc.), and hypoglycemia.

#### 2.1.5. Healthcare Providers

At present, collaborative glucose management by a nurse–physician team is widely adopted in existing studies. However, with the development of clinical pharmacy, pharmacists are gradually becoming involved in clinical practice, providing medication counseling and therapeutic management for patients and other professionals. It has been proven that pharmacists participating in glucose management can improve clinical outcomes [25–27]. To this end, we set the following three levels: independent management of nonendocrine department doctors, joint care of doctors and nurses, and doctor–nurse–pharmacist collaborative management.

#### 2.1.6. Management Cost

This attribute does not represent the actual treatment cost but serves as a reference indicator to assess patients' willingness to pay for glycemic control. It is measured in two levels: 2500 Chinese Yuan (CNY) and 5000 CNY.

After attributes and levels were determined, 10 hospitalized diabetes patients were inquired about their perceptions and expectations of antihyperglycemic therapy. We assessed if they can fully understand the meaning of the included content to ensure the smooth implementation of the subsequent survey.

### 2.2. Choice Question Format

Various attributes and different levels in Table 1 could form 486 (3 × 3 × 3 × 3 × 3 × 2) possible scenarios, which can be burdensome for respondents and result in lower completion rates and questionnaire quality. Therefore, we used OED to reduce the number of scenarios to a more feasible number while still being able to weigh the importance of all attribute levels and obtain 18 management patterns (refer to Table 2). We then selected a scenario with moderate levels for each attribute as comparison (i.e., Programme B) and paired it with other scenarios (i.e., Programme A) to create different question options (as shown in Table 3). Finally, a total of 17 choice questions were generated and were randomly divided into two questionnaires (Questionnaire 1: nine choice questions; Questionnaire 2: eight questions), aiming to alleviate respondent burden during the survey process.

We assessed the validity of responses by adding a test question at the end of questionnaires, in which Programme A outperformed Programme B in all six attribute levels. Respondents who chose Programme B were excluded from the final analysis as they did not fill in the questionnaire carefully or did not fully comprehend the contents.

Furthermore, each respondent was requested to provide their basic demographic information and disease condition. This is because physician decision-making behavior is not only influenced by treatment preference attributes but may also vary due to the differing personal characteristics of patients.

### 2.3. Respondents

Patients meeting the inclusion criteria were recruited: (1) diagnosis of T2DM according to the 2020 Chinese Diabetes Society Guideline [3]; (2) hospitalized in a nonendocrine department due to other comorbidities; (3) good understanding and communication skills, able to independently complete the questionnaire; and (4) signed informed consent form.

To determine the minimum sample size necessary for DCE surveys, we utilize the commonly employed “rules of thumb,” governed by the formula *N* > 500*c*/(*a* × *t*) [23, 28]. Here, “*c*” denotes the largest number of levels among all attributes, “*t*” signifies the number of choice questions in a questionnaire, and “*a*” represents the number of alternatives in one question. In our study, the attributes have a maximum of three levels, resulting in *c* = 3. Questionnaire 1 comprises nine questions, while Questionnaire 2 consists of eight questions, leading to *t* = 8 or 9. Additionally, each question offers two alternative patterns for choosing, thus *a* = 2. Utilizing *t* = 8 in the calculation, the required sample size is approximately 94. However, considering the importance of sample representativeness and accounting for a certain noncompletion rate, we ultimately surveyed 200 patients.

### 2.4. Statistical Analysis

Data were collected through face-to-face survey interviews. Trained investigators explained the purpose of the study and the contents of the questionnaire to eligible patients. Patients then independently filled out the questionnaire, and the completed questionnaires were collected on the spot. The data were entered into Microsoft Office Excel (2013) and subsequently analyzed using SPSS Statistics 25.0. The conditional logit (CL) model is the most commonly used model for DCE analysis which can explain both individual characteristics and those attributes and levels [19, 20]. In the CL model, the regression coefficients represent the magnitude and direction of preference for each attribute level. Larger positive coefficients suggest that patients prefer more of that attribute level, while smaller or negative coefficients indicate a lesser preference for that attribute level on average.

## 3. Results

### 3.1. Respondent Characteristics

A total of 200 T2DM patients hospitalized in nonendocrine departments were surveyed. Of these, 100 filled out Questionnaire 1, and the remaining 100 filled out Questionnaire 2. Finally, 157 (78.5%) valid questionnaires were returned. Among the unqualified questionnaires, 15 (7.5%) were incomplete, 28 (14.0%) failed the validity test question, and their data were excluded from the analysis.

The sociodemographic information and clinical characteristics of respondents are presented in Table 4. The mean age was 51.9 ± 12.30 years, with the majority being male (72.6%). The majority of the respondents were married (96.2%). In terms of cultural level, only 30 (19.1%) had a junior high school education or below, while 54.8% had a college degree or above. Regarding occupation, 63 (40.1%) were employed, 25 (15.9%) were workers, 15 (9.6%) were freelancers, and 38 (24.2%) were retired. Among the respondents, 17 people (10.8%) had an annual income of less than 50,000 CNY, 44 (28.0%) had an income of 50,000–100,000 CNY, 39 (24.8%) had an income of 100,000–150,000 CNY, 15 (9.6%) had an income of 150,000–250,000 CNY, and 7 (4.5%) had an income of more than 250,000 CNY. Most respondents had medical insurance, while only 10 people were self-paying, accounting for 6.4%. The diabetes duration ranged from 1 to 32 years (mean duration = 9.45 ± 7.54 years). Nearly half (46.5%) of the patients surveyed were diagnosed with diabetes based on clinical symptoms such as dry mouth, polydipsia, weight loss, and fatigue.

### 3.2. Analysis of the Attributes Influencing Patients' Choices of Hospital-Wide Glycemic Control Programme

**Figure 1:**
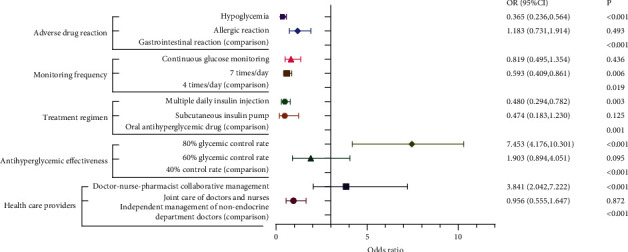
Regression analysis of the attributes influencing T2DM patients' choices of hospital-wide glycemic control programme.

Antihyperglycemic effectiveness, healthcare providers, treatment regimen, monitoring frequency, and adverse reaction were the five key attributes affecting patients' choices of an in-hospital glycemic control programme, while the management cost had no effect on their preferences. See Figure 1 and Table 5 for specific analysis results.

The different attributes had varying magnitudes and impacts on patient choices. The 80% glycemic control rate had the largest coefficient (*β* = 2.009, *p* < 0.001), indicating it had the greatest influence on patient preference. Additionally, patients favored a healthcare team that included clinical pharmacists (*β* = 1.346, *p* < 0.001) over nonendocrine department self-management. Regarding treatment regimens, patients were more likely to choose oral antihyperglycemic drugs, while subcutaneous insulin injection or insulin pump had a negative impact. Other factors that negatively affected patient preference included hypoglycemia (*β* = −1.008) and glucose monitoring 7 times a day (*β* = −0.523).

### 3.3. Analysis of Individual Characteristics Influencing Treatment Preferences of T2DM Patients

#### 3.3.1. Gender

As shown in Table 6, there were significant differences in the treatment preferences of hospitalized T2DM patients according to gender. Healthcare providers, antihyperglycemic effectiveness, monitoring frequency, and adverse reaction all influenced the choice of male patients, while only healthcare providers and monitoring frequency affected females. This difference may be attributed to the small sample size of our female respondents. Among male hospitalized patients, the coefficient of 80% glycemic control rate was the largest (*β* = 1.957, *p* < 0.001), followed by specialist collaborative management (*β* = 1.542, *p* < 0.001). This indicates that efficacy was the primary consideration for male patients when choosing an in-hospital glycemic control programme, while treatment regimen and monitoring frequency received less attention. In contrast, female respondents preferred a healthcare team that included clinical pharmacists and preferred fewer monitoring frequencies.

#### 3.3.2. Age

According to the analysis presented in Table 7, antihyperglycemic effectiveness was the primary concern for patients aged 65 and below, with the largest regression coefficient (*p* < 0.001). Among this group, patients aged 40 and below prioritized treatment regimens as their second concern, with a negative impact observed for those using insulin pumps. For patients aged 41–65, the second most important concern was medical service providers, with a preference for a stewardship group including clinical pharmacists (*p* < 0.001). Patients aged 66 years and above were primarily influenced by two attributes: medical service providers and glucose monitoring frequency. The coefficient for doctor–nurse–pharmacist collaborative management was 1.186 (*p* = 0.001), while glucose monitoring 7 times a day had a negative impact with a coefficient of −0.703 (*p* = 0.024).

#### 3.3.3. Diabetes Duration

The duration of diabetes was found to have an impact on patients' treatment attribute preferences. As shown in Table 8, among respondents with a disease course ≤1 year, only antihyperglycemic effectiveness had a significant impact, with the regression coefficient of 80% glycemic control rate being 1.510 (*p* < 0.001). For patients with a disease course of 2–5 years, in addition to efficacy, treatment regimen and adverse reaction also affected their preferences, but the coefficient of 80% control rate remained the largest (*β* = 2.699, *p* < 0.001). Patients in this group also had a significant aversion to the use of insulin subcutaneous pumps, as indicated by a negative coefficient of −2.021 (*p* <0.001) when compared with oral drugs. For patients with a disease course of 6–10 years, doctor–nurse–pharmacist collaborative management had the greatest impact on their treatment preferences (*β* = 2.225, *p* < 0.001). Finally, for patients with a disease course >10 years, the attributes that affected their treatment preferences increased, including medical service providers, antihyperglycemic effectiveness, treatment regimen, and adverse reaction, and the coefficient of treatment regimens was the largest (*p* < 0.001).

#### 3.3.4. Education Background

The analysis results presented in Table 9 demonstrate that the factors influencing the decision-making behavior of patients vary based on their level of education. Patients with a junior high school education or below were found to not be affected by the attributes included in the study. For patients with a high school to undergraduate education, the primary factor influencing their choice was treatment effectiveness (*p* < 0.001). Among them, college-educated patients also considered monitoring frequency, while those with a bachelor's degree were secondly concerned with the treatment regimen and rejected the use of insulin. Additionally, patients with a postgraduate education or above were found to prioritize medical service providers and glucose monitoring frequency. They exhibited a preference for management by a doctor–nurse–pharmacist collaborative team (*β* = 2.197, *p* = 0.003) and CGM, which differed from the preferences of patients with lower educational levels.

#### 3.3.5. Income

Patients with an annual income of less than 150,000 CNY primarily prioritize antihyperglycemic effectiveness. Among them, for patients with an annual income of less than 50,000 CNY, another attribute that influenced their choice was the treatment regimen, with a coefficient of −1.163 (*p* = 0.005) for using an insulin subcutaneous pump. Patients with an annual income between 60,000 and 100,000 CNY prioritize antihyperglycemic effectiveness first, followed by medical service providers, management costs, adverse reactions, and finally glucose monitoring frequency. For those with an income level of 110,000–150,000 CNY, the influential attributes were glycemic control rate, medical service provider, and treatment regimen. None of the included attributes had an effect on patients with an annual income of more than 150,000 CNY, possibly due to the small sample size of this group. See Table 10 for details.

## 4. Discussion

DCE is a quantitative technique used to capture individuals' preferences based on Lancaster's economic theory [29]. According to this theory, goods and services can be defined by their essential characteristics, known as attributes and levels. The value an individual places on a good or service is derived from the combination of these characteristics. In this study, patients were asked to choose their preferred in-hospital glycemic control programme from several hypothetical hyperglycemia management scenarios described by six attributes and 17 corresponding levels. The results revealed that antihyperglycemic effectiveness, healthcare providers, treatment regimen, monitoring frequency, and adverse reactions had a significant impact on the treatment preferences of hospitalized T2DM patients. The magnitude and direction of the regression coefficients represented differences in the degree of influence of these attributes on patients. Notably, the coefficient of antihyperglycemic effectiveness was the largest, indicating that patients highly valued treatment efficacy, which is consistent with the conclusion of another DCE study on the preferences for antihyperglycemic drugs among Chinese T2DM patients [20]. However, there was no significant difference when respondents chose the glycemic control rate of 40% and 60%, which indicated that patients may lower some efficacy expectations in exchange for better effects of other attributes when there is little difference in the level of efficacy.

We also found that patients prefer being managed by a multidisciplinary team composed of endocrinologists, clinical pharmacists, and nurses rather than by department doctors alone. With the specialized development of modern medicine, physicians often feel inadequate in diagnosing and treating diseases beyond their own specialty due to a lack of equipment and therapy experience.

Hyperglycemia in hospitalized patients can have multiple causes. It may be due to preexisting pancreatic dysfunction or insulin resistance. Additionally, the stress response in acute illnesses such as trauma, burns, sepsis, and postoperative conditions can lead to transient hyperglycemia [11]. Furthermore, the use of glucocorticoids can cause great fluctuations in blood glucose concentration, even in patients who had good glycemic control prior to admission. For patients experiencing dysglycemia in nonendocrine departments, collaborative management by a specialist team is even more necessary, as it can provide them with professional diagnosis and treatment plans, as well as guidance on drugs, diet, exercise, and diabetes care in daily life. Our findings are consistent with those of Mühlbacher et al., who identified management cost, trust and respect, multidisciplinary care, and SDM as critical attributes influencing patient preferences [30]. These results highlight the significance of effective doctor–patient communication and patient-centered care.

In terms of the choice of treatment regimens, the Chinese Diabetes Society guideline and expert consensus on glycemic control of hospitalized patients have fully acknowledged the significant role of insulin therapy and recommend it as the preferred option [3, 24]. However, this study found that patients' acceptance of insulin was not high when compared to oral drugs. Previous research has indicated that a patient's previous experience with insulin significantly influences their preference for different treatment options. Patients who administer insulin for the first time may be apprehensive, but considering its antihyperglycemic effectiveness, they still prefer insulin regimens, while those with a history of insulin use prefer oral drugs [31]. Therefore, it is crucial for clinical pharmacists to engage in comprehensive communication with patients and educate them about the purpose and advantages of insulin therapy to improve treatment adherence.

Currently, capillary blood glucose monitoring is the most commonly used method in clinical practice, despite being painful and invasive. The study results show that as the monitoring frequency increased, the willingness of patients to choose this option gradually decreased. Although CGM is less invasive, our respondents were less likely to choose this approach, probably due to its high cost and patients' limited knowledge. In the actual management process, an individualized monitoring plan should be formulated based on the patient's blood glucose concentration, diet, and exercise status. However, judging from the regression coefficient of this attribute, the absolute value of each level was small, indicating that it has a minor impact on patient choices. Therefore, while monitoring frequency is still an important consideration, it should not be prioritized at the expense of better levels of other key attributes.

In previous studies, the effectiveness of treatment and the occurrence of hypoglycemic symptoms were often considered together to determine their relative importance [19, 20]. It has been found that, compared to hypoglycemic events, the effect of glycemic control is more likely to influence patient treatment preferences [20]. This conclusion is consistent with the results of the current study. Among the three types of adverse events considered, hypoglycemia had the greatest impact on patient choice, followed by gastrointestinal reactions. Allergic reactions, such as skin itching, were considered the most acceptable, likely due to their relatively low incidence. Patients may choose to tolerate allergic reactions to avoid serious hypoglycemia or gastrointestinal reactions. To enhance the quality of in-hospital glucose management, clinical pharmacists should focus on monitoring and addressing drug-related adverse events. Implementing preventive measures and promptly identifying and managing these events can significantly improve patient experiences and treatment adherence.

Additionally, we examined the influence of different personal characteristics on the preferences for the glycemic control programme. We found that there were significant differences based on gender. Among male respondents, the relative importance of attributes was the same as that of the entire sample, with antihyperglycemic effectiveness being the primary concern. In contrast, female respondents placed greater emphasis on the collaborative management provided by doctor–nurse–pharmacist multidisciplinary teams and preferred CGM. This comparison highlights that male patients tend to prioritize treatment effect, while female patients prioritize treatment comfort and minimizing pain experience. These gender-specific preferences should be taken into account when developing individualized treatment plans.

The attributes that patient groups of different ages and disease courses preferred were also different. Young and newly diagnosed diabetes patients focused more on the effect of glycemic control, possibly because of insufficient awareness of their disease and a desire to achieve healthy glucose concentration in a short time. As disease duration increased, patients gradually realized that diabetes is a multifactorial metabolic disorder that requires long-term and comprehensive management, so patients over 66 years old and those with a history of more than 10 years turned to paying more attention to multidisciplinary team stewardship.

In this study, among patients with an education level of junior high school and below, the included attributes did not affect their choice behavior, which might be because their limited knowledge made it difficult for them to make reasonable choices. In this scenario, medical staff should have full communication with the patients to enhance their understanding of diabetes. For patients with a bachelor's degree or below, antihyperglycemic effectiveness was the primary attribute of concern. In contrast, respondents with postgraduate education and above were more inclined to specialist team management; additionally, their preference for CGM was also significantly different from that of patients with other academic qualifications, potentially because of relatively good recognition of their illness.

Finally, patients across different income levels all prioritize antihyperglycemic effectiveness, but the degree of its influence on their choices varies. The magnitude of the regression coefficient indicates that patients with higher annual income levels place greater emphasis on glycemic control rates. Furthermore, compared to low-income patients, those with middle to high incomes tend to prefer collaborative management by multidisciplinary teams. It is evident that as economic status improves, people increasingly value a comfortable lifestyle and an ideal state of health, thus placing more emphasis on treatment effects.

This study has several limitations.

Firstly, DCE research has inherent limitations. While it has the advantage of simulating real-world situations and capturing patients' choices more realistically compared to simple rating scale exercises, it relies on participants making hypothetical choices rather than observed choices. SPs in hypothetical scenarios may not fully reflect the complexities and trade-offs that patients consider in real-life decision-making, which involves emotional, financial, and clinical factors with real consequences [32]. This limitation is common to all SP measurement methods. However, some studies propose that employing two or more measurement methods simultaneously can provide a comprehensive understanding of patient preferences and serve as a test of convergent validity [33]. For instance, best–worst scaling (BWS) is another widely used method for measuring SPs, where participants select the “best” and “worst” alternatives from choice tasks comprising at least three options. Systematic reviews indicate that both methods may yield similar validity, although there may be variations in elicited preferences [34]. The choice between methods is likely influenced by normative considerations regarding coherence with theoretical frameworks and pragmatic factors related to ease of data collection. Nonetheless, employing both methods together can undoubtedly help mitigate biases inherent in survey methodologies.

Another limitation is the potential impact of respondents' insufficient understanding of the included attributes, levels, and choice tasks on the quality or validity of the resulting preference data [35]. Despite the researchers providing detailed explanations of the study's purpose and filling instructions, as well as conducting a pilot test to ensure clarity and comprehension of technical terms like “glycemic control rate,” during the formal experiment, 14.0% of respondents still failed the validity test question. This highlights the importance of effective communication with patients in future research, particularly with older individuals who may have reduced cognitive abilities or individuals with lower levels of cultural literacy.

Furthermore, in determining the attributes and levels for this study, we followed the guidance provided by the ISPOR task force [36]. Considering that too many attribute levels could burden respondents and require a larger sample size, we chose to include only the six most relevant features related to the in-hospital glycemic control programme and limit each attribute to two or three levels. We had to make some trade-offs and exclude certain levels. For example, under the attribute “adverse drug reaction,” we omitted weight gain caused by sulfonylureas and glinides. This decision was based on the consideration that patients' weight might not fluctuate significantly during the short hospitalization period, and therefore, the importance of weight gain might not be as high as that of hypoglycemic events. However, when investigating long-term medication preferences after discharge, weight gain should be considered as a key characteristic.

Additionally, the study was a single-center cross-sectional survey with a limited sample size, which may restrict the generalizability of the findings to populations or healthcare facilities in different socioeconomic regions. For example, individuals in economically developed regions in eastern China may prioritize treatment effectiveness over management cost, being more willing to allocate additional medical expenses for better health outcomes. Similarly, in grassroots healthcare facilities lacking multidisciplinary teamwork (comprising clinical medicine, nursing, and clinical pharmacy), patients may exhibit less sensitivity to the healthcare provider attribute, having not experienced the benefits of coordinated multidisciplinary care. Future research endeavors will require an expansion of our sample size and collaboration with healthcare institutions across different regions and levels. This approach will enable a more comprehensive exploration of treatment preferences among diverse populations. The ultimate goal is to refine and optimize the existing hospital-wide glycemic management programme.

Finally, the study focused solely on the perspectives of patients and did not consider the viewpoints of other stakeholders, such as healthcare providers or payers involved in the in-hospital glycemic control programme. The inclusion of these perspectives could provide valuable insights into the feasibility and implementation of different treatment options. It is crucial to consider these limitations when interpreting the results of this study and when considering the implications for clinical practice and policy-making.

## 5. Conclusion and Practice Implications

This study employed the DCE method to analyze treatment preferences of T2DM patients hospitalized and admitted to nonendocrinology departments for a hospital-wide glycemic control programme. Antihyperglycemic effectiveness, healthcare providers, treatment regimen, monitoring frequency, and adverse reactions are the five key attributes influencing patient preference. In addition, the study revealed that patients with different personal characteristics have different treatment preferences. It is important for healthcare providers to consider these treatment preferences in order to ensure patient-centered hyperglycemia management.

Measuring patient preferences is a critical step, but it is just the beginning; these preferences must be effectively translated into actionable strategies within clinical practice. To achieve this goal, our team has devised a comprehensive plan outlining specific steps for enhancing our hospital-wide glycemic management programme. We have established a multidisciplinary whole-hospital blood glucose management team, harnessing the unique strengths of each discipline to deliver comprehensive diagnostic and treatment services tailored to individual patient needs. We are dedicated to conducting regular training sessions for our entire glycemic management team, with a focus on integrating patients' unique characteristics, treatment preferences, and the latest evidence-based medicine to craft personalized glycemic control plans, aiming to optimize antihyperglycemic effectiveness as the primary concern for patients. We also recognize the importance of patient education and provide patients with relevant information about diabetes medications during their hospitalization, enhancing their understanding and acceptance of glycemic-lowering medications, with a particular emphasis on insulin injections, thus fostering improved treatment adherence. Moreover, we will rigorously implement and monitor the hospital-wide glycemic management programme, conducting regular assessments of its cost, glycemic-lowering effects, and overall health benefits to ensure its cost-effectiveness and sustainability. Finally, our commitment to continuous improvement will drive us to refine the glycemic management programme further, taking into account not only patient preferences but also the preferences of other stakeholders and the programme's cost-effectiveness. Through these concerted efforts, we aim to translate our research findings into tangible strategies that will enhance hyperglycemia management in clinical practice and improve patient outcomes.

## Figures and Tables

**Table 1:** Attributes, levels, introduction, and assignments of the discrete choice experiment.

**Attributes**	**Introduction to patients**	**Levels**	**Assignments**
Antihyperglycemic effectiveness	This is about how well the treatment you receive in the hospital helps bring your blood sugar levels back to normal.	Glycemic control rate of high (80%)	0
		Glycemic control rate of moderate (60%)	1
		Glycemic control rate of low (40%)	2

Treatment regimen	This is about which type of glucose-lowering treatment you prefer—whether you prefer oral medication, injections, or using an insulin pump.	Oral antihyperglycemic drug	0
		Multiple daily insulin injections	1
		Subcutaneous insulin pump	2

Monitoring frequency	This tells you how often your blood sugar levels will be checked, so your doctor can better understand your condition.	4 times/day	0
		7 times/day	1
		Continuous glucose monitoring	2

Adverse drug reaction	This encompasses potential side effects of the treatment, informing you of any discomfort you may experience during the treatment process.	Gastrointestinal reaction	0
		Allergic reaction (skin pruritus, etc.)	1
		Hypoglycemia	2

Healthcare providers	These are the medical professionals who are responsible for providing you with medical care, so you know who you will be collaborating with.	Doctor–nurse–pharmacist collaborative management	0
		Joint care of doctors and nurses	1
		Independent management of nonendocrine department doctors	2

Management cost	This is about how much you are willing to pay for glucose-lowering treatment, not the actual price of the plan.	2500 CNY	0
		5000 CNY	1

**Table 2:** The 18 most representative alternative hypothetical scenarios selected from all possible combinations of attribute levels.

**Programme**	**Antihyperglycemic effectiveness**	**Treatment regimen**	**Monitoring frequency**	**Adverse drug reaction**	**Healthcare providers**	**Management cost**
1	0	2	2	2	2	1
2	1	2	2	0	2	1
3	0	0	0	0	0	0
4	0	1	0	1	2	1
5	0	1	1	0	1	1
6	2	0	2	1	1	0
7	2	0	1	0	2	0
8	1	1	2	1	0	0
9	1	2	0	0	1	0
10	2	1	2	0	0	1
11^a^	1	1	1	2	1	0
12	1	0	1	1	2	1
13	2	2	0	1	1	1
14	2	2	1	1	0	0
15	1	0	0	2	0	1
16	2	1	0	2	2	0
17	2	0	2	2	1	0
18	0	2	1	2	0	0

^a^This programme, which consists of moderate levels for each attribute, was selected as the comparison. The remaining alternatives were paired with it to form 17 choice questions.

**Table 3:** Example of question options.

**Attributes**	**Programme A**	**Programme B**
Healthcare providers	Independent management of nonendocrine department doctors	Joint care of doctors and nurses
Antihyperglycemic effectiveness	High glycemic control rate (80%)	Moderate glycemic control rate (60%)
Treatment regimen	Subcutaneous insulin pump	Multiple daily insulin injections
Monitoring frequency	Continuous glucose monitoring	7 times/day
Adverse drug reaction	Hypoglycemia	Hypoglycemia
Management cost	5000 CNY	2500 CNY
Your preference		

**Table 4:** Demographic and sociological characteristics of respondents.

**Individual characteristics**	**Classification**	**Amount (** **n** **)**	**Proportion (%)**
Gender	Male	114	72.6
	Female	43	27.4

Age	≤40 years	30	19.1
	41–65 years	102	64.9
	≥66 years	25	16

Marital status	Married	151	96.2
	Others	6	3.8

Education background	Junior high school and below	30	19.1
	High school	42	26.8
	Junior college	30	19.1
	Undergraduate	49	31.2
	Postgraduate and above	7	4.5

Occupation	Employee	63	40.1
	Worker	25	15.9
	Retired	38	24.2
	Freelancer	15	9.6
	Civil servant	16	10.2

Annual income (1000 CNY)	≤5	17	10.8
	60–100	44	28
	110–150	39	24.8
	160–250	15	9.6
	≥260	7	4.5

Medical insurance	Urban resident essential medical insurance	40	25.5
	Urban employee essential medical insurance	99	63.1
	New rural cooperative medical insurance	8	5.1
	Self-pay	10	6.4

Diabetes duration (year)	≤1	21	13.4
	2–5	45	28.7
	6–10	34	21.7
	>10	57	36.3

The way diabetes was first identified	Clinical symptoms	73	46.5
	Physical examination	60	38.2
	Unexpected discovery	24	15.3

**Table 5:** Regression analysis of the attributes influencing T2DM patients' choices of hospital-wide glycemic control programme.

**Attributes**	**Levels**	**β**	**SE**	**Wald**	**p**	**OR (95% CI)**
Healthcare providers	Independent management of nonendocrine department doctors (comparison)			18.878	<0.001	
	Joint care of doctors and nurses	−0.045	0.278	0.026	0.872	0.956 (0.555, 1.647)
	Doctor–nurse–pharmacist collaborative management	1.346	0.322	17.439	<0.001	3.841 (2.042, 7.222)

Antihyperglycemic effectiveness	40% glycemic control rate (comparison)			57.432	<0.001	
	60% glycemic control rate	0.643	0.386	2.784	0.095	1.903 (0.894, 4.051)
	80% glycemic control rate	2.009	0.296	46.201	<0.001	7.453 (4.176, 10.301)

Treatment regimen	Oral antihyperglycemic drug (comparison)			13.071	0.001	
	Multiple daily insulin injections	−0.735	0.249	8.692	0.003	0.480 (0.294, 0.782)
	Subcutaneous insulin pump	−0.746	0.486	2.354	0.125	0.474 (0.183, 1.230)

Monitoring frequency	4 times/day (comparison)			7.920	0.019	
	7 times/day	−0.523	0.190	7.565	0.006	0.593 (0.409, 0.861)
	Continuous glucose monitoring	−0.200	0.257	0.608	0.436	0.819 (0.495, 1.354)

Adverse drug reaction	Gastrointestinal reaction (comparison)			22.282	<0.001	
	Allergic reaction	0.168	0.245	0.470	0.493	1.183 (0.731, 1.914)
	Hypoglycemia	−1.008	0.223	20.516	<0.001	0.365 (0.236, 0.564)

Management cost	2500 CNY	—	—	—	—	—

**Table 6:** Regression analysis of patients by different genders.

**Attributes**	**Levels**	**Male**		**Female**	
		**β**	**p**	**β**	**p**
Healthcare providers	Independent management of nonendocrine department doctors (comparison)	—	—	—	—
	Joint care of doctors and nurses	−0.280	0.392	−0.211	0.084
	Doctor–nurse–pharmacist collaborative management	1.542	<0.001	1.172	<0.001

Antihyperglycemic effectiveness	40% glycemic control rate (comparison)	—	—	—	—
	60% glycemic control rate	0.257	0.581	—	—
	80% glycemic control rate	1.957	<0.001	—	—

Treatment regimen	Oral antihyperglycemic drug (comparison)	—	—		
	Multiple daily insulin injections	−0.506	0.084	—	—
	Subcutaneous insulin pump	−0.323	0.573	—	—

Monitoring frequency	4 times/day (comparison)	—	—	—	—
	7 times/day	−0.550	0.014	−0.347	0.113
	Continuous glucose monitoring	−0.506	0.224	0.876	0.004

Adverse drug reaction	Gastrointestinal reaction (comparison)	—	—	—	—
	Allergic reaction	0.343	0.261	—	—
	Hypoglycemia	−0.901	0.001	—	—

Management cost	2500 CNY (comparison)	—	—	—	—

**Table 7:** Regression analysis of patients by age stratification.

**Attributes**	**Levels**	**≤40** **years**		**41–65** **years**		**≥66** **years**	
		**β**	**p**	**β**	**p**	**β**	**p**
Healthcare providers	Independent management of nonendocrine department doctors (comparison)	—	—	—	—	—	—
	Joint care of doctors and nurses	—	—	0.181	0.555	0.317	0.278
	Doctor–nurse–pharmacist collaborative management	—	—	1.190	<0.001	1.186	0.001

Antihyperglycemic effectiveness	40% glycemic control rate (comparison)	—	—	—	—	—	—
	60% glycemic control rate	1.605	0.001	0.550	0.206	—	—
	80% glycemic control rate	3.330	<0.001	1.760	<0.001	—	—

Treatment regimen	Oral antihyperglycemic drug (comparison)	—	—	—	—	—	—
	Multiple daily insulin injections	−1.189	0.004	−0.865	0.001	—	—
	Subcutaneous insulin pump	−2.108	<0.001	−1.125	0.023	—	—

Monitoring frequency	4 times/day (comparison)	—	—	—	—	—	—
	7 times/day	—	—	—	—	−0.703	0.024
	Continuous glucose monitoring	—	—	—	—	0.269	0.476

Adverse drug reaction	Gastrointestinal reaction (comparison)	—	—	—	—	—	—
	Allergic reaction	−0.097	0.770	−0.074	0.748	—	—
	Hypoglycemia	−1.345	0.006	−0.862	0.001	—	—

Management cost	2500 CNY (comparison)	—	—	—	—	—	—

**Table 8:** Regression analysis of patients with different diabetes duration.

**Attributes**	**Levels**	**≤1** **year**		**2–5 years**		**6–10 years**		**>10 years**	
		**β**	**p**	**β**	**p**	**β**	**p**	**β**	**p**
Healthcare providers	Independent management of nonendocrine department doctors (comparison)	—	—	—	—	—	—	—	—
	Joint care of doctors and nurses	—	—	—	—	0.072	0.797	1.098	0.010
	Doctor–nurse–pharmacist collaborative management	—	—	—	—	2.225	<0.001	0.131	0.758

Antihyperglycemic effectiveness	40% glycemic control rate (comparison)	—	—	—	—	—	—	—	—
	60% glycemic control rate	0.234	0.554	1.483	<0.001	0.682	0.019	2.657	<0.001
	80% glycemic control rate	1.510	<0.001	2.699	<0.001	0.728	0.009	3.075	<0.001

Treatment regimen	Oral antihyperglycemic drug (comparison)	—	—	—	—	—	—	—	—
	Multiple daily insulin injections	—	—	−1.409	<0.001	—	—	−1.783	<0.001
	Subcutaneous insulin pump	—	—	−2.021	<0.001	—	—	−3.207	<0.001

Monitoring frequency	4 times/day (comparison)	—	—	—	—	—	—	—	—
	7 times/day	—	—	—	—	—	—	—	—
	Continuous glucose monitoring	—	—	—	—	—	—	—	—

Adverse drug reaction	Gastrointestinal reaction (comparison)	—	—	—	—	—	—	—	—
	Allergic reaction	—	—	0.088	0.727	—	—	−0.862	0.006
	Hypoglycemia	—	—	−1.134	0.002	—	—	−1.923	<0.001

Management cost	2500 CNY (comparison)	—	—	—	—	—	—	—	—

**Table 9:** Regression analysis of patients with different education levels.

**Attributes**	**Levels**	**Junior high school and below**		**High school**		**Junior college**		**Undergraduate**		**Postgraduate and above**	
		**β**	**p**	**β**	**p**	**β**	**p**	**β**	**p**	**β**	**p**
Healthcare providers	Independent management of nonendocrine department doctors (comparison)	—	—	—	—	—	—	—	—	—	—
	Joint care of doctors and nurses	—	—	—	—	—	—	—	—	−0.716	0.179
	Doctor–nurse–pharmacist collaborative management	—	—	—	—	—	—	—	—	2.197	0.003

Antihyperglycemic effectiveness	40% glycemic control rate (comparison)	—	—	—	—	—	—	—	—	—	—
	60% glycemic control rate	—	—	−0.031	0.907	0.558	0.152	1.986	<0.001	—	—
	80% glycemic control rate	—	—	1.962	<0.001	1.574	<0.001	3.117	<0.001	—	—

Treatment regimen	Oral antihyperglycemic drug (comparison)	—	—	—	—	—	—	—	—	—	—
	Multiple daily insulin injections	—	—	—	—	—	—	−1.552	<0.001	—	—
	Subcutaneous insulin pump	—	—	—	—	—	—	−1.681	<0.001	—	—

Monitoring frequency	4 times/day (comparison)	—	—	—	—	—	—	—	—	—	—
	7 times/day	—	—	—	—	−0.824	0.032	—	—	0.142	0.790
	Continuous glucose monitoring	—	—	—	—	0.448	0.223	—	—	1.983	0.031

Adverse drug reaction	Gastrointestinal reaction (comparison)	—	—	—	—	—	—	—	—	—	—
	Allergic reaction	—	—	—	—	—	—	−0.728	0.004		
	Hypoglycemia	—	—	—	—	—	—	−1.323	<0.001		

Management cost	2500 CNY (comparison)	—	—	—	—	—	—	—	—		

**Table 10:** Regression analysis of patients with different annual income levels.

**Attributes**	**Levels**	**≤50,000 CNY**		**60,000–100,000 CNY**		**110,000–150,000 CNY**		**160,000–250,000 CNY**		**≥260,000 CNY**	
		**β**	**p**	**β**	**p**	**β**	**p**	**β**	**p**	**β**	**p**
Healthcare providers	Independent management of nonendocrine department doctors (comparison)	—	—	—	—	—	—	—	—	—	—
	Joint care of doctors and nurses	—	—	−0.464	0.120	0.572	0.240	—	—	—	—
	Doctor–nurse–pharmacist collaborative management	—	—	1.359	0.002	1.631	0.001	—	—	—	—

Antihyperglycemic effectiveness	40% glycemic control rate (comparison)	—	—	—	—	—	—	—	—	—	—
	60% glycemic control rate	−0.228	0.024	0.428	0.343	0.215	0.685	—	—	—	—
	80% glycemic control rate	1.414	<0.001	1.810	<0.001	1.940	<0.001	—	—	—	—

Treatment regimen	Oral antihyperglycemic drug (comparison)	—	—	—	—	—	—	—	—	—	—
	Multiple daily insulin injections	−0.261	0.303	—	—	−1.323	0.004	—	—	—	—
	Subcutaneous insulin pump	−1.163	0.005	—	—	−1.437	0.084	—	—	—	—

Monitoring frequency	4 times/day (comparison)	—	—	—	—	—	—	—	—	—	—
	7 times/day	—	—	−0.814	0.018	—	—	—	—	—	—
	Continuous glucose monitoring	—	—	0.153	0.710	—	—	—	—	—	—

Adverse drug reaction	Gastrointestinal reaction (comparison)	—	—	—	—	—	—	—	—	—	—
	Allergic reaction	—	—	0.309	0.351	—	—	—	—	—	—
	Hypoglycemia	—	—	−0.843	0.038	—	—	—	—	—	—

Management cost	2500 CNY (comparison)	—	—	−0.881	0.017	—	—	—	—	—	—

## Data Availability

The datasets analyzed in this study, including patient characteristic information and preference questionnaires, are subject to privacy and ethical considerations. In order to protect patient confidentiality, they are not publicly available.
